# Prevalence of *algD*, *pslD*, and *pelF* Genes Involved in Biofilm *Formation in Clinical MDR Pseudomonas aeruginosa* Strains

**DOI:** 10.30699/ijp.2025.2057524.3439

**Published:** 2025-08-15

**Authors:** Zahra Haghighatian, Negar Shirani, Gholamreza Goudarzi, Pegah Shakib, Fatemeh Jahani, Sodabeh Zare

**Affiliations:** 1 Department of Pathology, Faculty of Medicine, Lorestan University of Medical Sciences, Khorramabad, Iran; 2 Student Research Committee, Lorestan University of Medical Sciences, Khorramabad, Iran; 3 Department of Microbiology, School of Medicine, Lorestan University of Medical Sciences, Khorramabad, Iran; 4 Razi Herbal Medicines Research Center, Lorestan University of Medical Sciences, Khorramabad, Iran; 5 Department of Biology, Khorramabad Branch, Islamic Azad University, Khorramabad, Iran; 6 Department of Biostatistics and Epidemiology, School of Health and Nutrition, Lorestan University of Medical Sciences, Khorramabad, Iran

**Keywords:** Pseudomonas aeruginosa, Biofilm, Multidrug resistance (MDR), Exopolysaccharides

## Abstract

**Background & Objective:**

The purpose of this research was to determine the frequency of *algD, pslD, *and* pelF genes* in biofilm formation among MDR and non-MDR clinical strains of *Pseudomonas aeruginosa* in Khorramabad, Iran (2024).

**Methods:**

This cross-sectional study included all *Pseudomonas aeruginosa* isolates collected from various clinical samples in Khorramabad teaching hospitals in 2024. After confirming the isolates and determining their antibiotic resistance patterns using the disc diffusion method according to the Clinical and Laboratory Standards Institute (CLSI) guidelines, *algD, pelF,* and *pslD* genes were detected by PCR.

**Results:**

The highest sensitivity was observed to imipenem (75%) and meropenem (71.3%), while the greatest resistance was recorded against ciprofloxacin, ceftazidime, and tobramycin 45 (56.25%). The frequencies of the *algD, pelF*, and *pslD* genes were 88.8, 76.3, and 96.3%, respectively. A significant association was found between the *PelF* and *algD* genes with multidrug resistance (MDR) (P<0.05).

**Conclusion:**

The presence of multi-drug resistance (MDR) in this study indicates the need for serious measures to control infections caused by this bacterium. Further research is recommended to explore the contribution of biofilm-associated genes to the development of multi-drug resistance (MDR).

## Introduction

Nonalcoholic fatty liver disease (NAFLD) is now one of *Pseudomonas aeruginosa* is a flagellated, Gram-negative bacilli belonging to the Pseudomonas genus. It is an opportunistic organism that poses a major risk to patients with burns and cancer and causes hospital infections (1,2). This bacterium causes bacteremia, pneumonia, septicemia and Intensive care unit (ICU) infections. The prevalence of antibiotic resistance in *Pseudomonas aeruginosa* is increasing, with more than 10% of *Pseudomonas aeruginosa* isolated around the world are multi-drug resistant (MDR) strains (3,4).* Pseudomonas aeruginosa* has multiple virulence factors, which include structural components (polysaccharide pill, capsule, lipopolysaccharide, pyocyanin toxin) and various enzymes (exotoxin A, cytotoxin, elastase, alkaline protease, phospholipase C) (5-8).

In many bacteria, such as *Pseudomonas aeruginosa*, biofilm production causes resistance to antimicrobial agents and can eventually lead to the spread of strains resistant to several drugs. Bacterial cells are shielded from the negative effects of drugs and the host's immunological response inside the biofilm matrix (9). Exopolysaccharides (EPS), proteins, and extracellular DNA (eDNA) make up the majority of the biofilm matrix. Alginate, Psl, and Pel are three significant exopolysaccharides that *Pseudomonas aeruginosa* synthesizes and secretes (10, 11). 13 genes make up the production route for alginate, which is composed of mannuronic and glucuronic acids included *AlgE, AlgK, Alg44, Alg8, D, C, A, F, J, I, L, X, and G* (12, 13).

This study aims to investigate the genes associated with biofilm formation, specifically *algD, pslD, and pelF,* as well as the antibiotic resistance patterns of *Pseudomonas aeruginosa* isolated from clinical samples in Khorramabad, Iran (2024), given the high prevalence of hospital infections caused by this pathogen and the emergence of antibiotic resistance.

## Materials and methods

### Isolation and Identification of Pseudomonas aeruginosa strains

In the cross-sectional study, isolation of the *Pseudomonas aeruginosa* strains from clinical samples performed in educational hospitals in Khorramabad City, western Iran from February to July 2024. Then, the collected *Pseudomonas aeruginosa* strains were confirmed by standard microbiological tests including sugar consumption, examination of oxidase, catalase, urease, IMVIC *(*Indole, Methyl red, Voges-Proskauer, Citrate)*.*

The information related to the samples, including the age, sex of the patients, and the type of sample was recorded.

### Antimicrobial susceptibility testing

The sensitivity and resistance pattern to antibiotics were assessed using the Kirby-Bauer test with the disc diffusion method according to the Clinical and Laboratory Standards Institute (CLSI) guidelines (14). To detection of the antibiotic resistance pattern of strains were used 11 antibiotics including ceftazidime (30µg), ciprofloxacin(5µg), cefepime (30µg), imipenem (10µg), amikacin(30µg), gentamicin (10µg), levofloxacin(5µg), piperacillin(100µg), piperacillin/ tazobactam (100/10µg), meropenem (10µg), and tobramycin (10µg), (Rosco, Denmark). After overnight incubation of test strains on Mueller Hinton Agar medium (Merck, Germany) plates, the diameter of Zone of Inhibition (ZOI) of disks were measured and recorded in millimeter. Strains resistant to three or more antibiotic classes were defined as multidrug-resistant (MDR) (15).

### Crystal violet Microtiter plate biofilm production assay

To perform this test, the strains were cultured in LB broth (Merck, Germany) and then 100 μl of these strains were re-cultured in 96-well plates and incubated for 24 hours at 37°C. After washing the wells several times with water, the wells were stained with 0.2% crystal violet for 15 minutes. After washing the plates several times and removing excess dye with distilled water, 200 μl of 96% ethanol was added to the wells of the plate and then 125 μl of this mixture was transferred to the wells of another 96-well plate, then the absorbance reading was performed at 590 nm (A590) (16). *Pseudomonas aeruginosa* ATCC27853, obtained from the National Center for Genetic and Biological Resources of Iran, was used as a positive control.

### DNA extraction and tracking of algD, pslD, pelF genes involved in biofilm

DNA extraction of the isolates was done using Sinna Pure DNA kit (cat. NO. PR881613) prepared by Sina Clone Company of Iran based on the instructions in the kit. Then the genes involved in the biofilm *algD, pslD, pelF* were detected in *Pseudomonas aeruginosa* isolates with specific primers (Table 1). The temperature program used to perform PCR is shown in Table 1.

### Statistical analysis

Statistical data analysis was conducted using SPSS version 21 software. In order to analyze the data, descriptive statistics (frequency, percentage, average) were implemented. The chi-square test or Fisher's exact test was implemented to investigate the correlation between the frequency of genes implicated in biofilm formation and variables. A significance level of less than 0.05 was taken into account.

**Table 1:** The sequence of primers used in our study

Ref	Thermocycler programs	Size (bp)	Sequences (5´ to 3´)	Genes
(17)	30 cycles (Denaturation:94 °C for 1 min, Annealing: 58 °C for 1min, Extension: 72°C for 1min), final Extension: 72°C for 7 min	593	F-CTACATCGAGACCGTCTGCC R-GCATCAACGAACCGAGCATC	*algD*
		789	F-GAGGTCAGCTACATCCGTCG R-TCATGCAATCTCCGTGGCTT	*pelF*
	Initial denaturation: 94 °C for 5 min.30 cycles (Denaturation:94 °C for 30s, Annealing: 56 °C for 30s, Extension: 72°C for 30s), final Extension: 72°C for 7 min	369	F-TGTACACCGTGCTCAACGAC R-CTTCCGGCCCGATCTTCATC	*pslD*

## Results

### Bacterial isolates and antimicrobial susceptibility testing

Out of 80 *Pseudomonas aeruginosa* clinical isolates,46 (57.5%) of them obtained from men and the other 34(52.5%) obtained from women. 79 (98.8%) acquired from hospitalized patients and 1(2.2%) acquired from out-patients. 36 isolates (45%) were isolated from urine, 22 samples (27.5%) from sputum,12 (15%) from wounds, 4(5%) from Secretions, and 6(7.5%) from blood samples. The average age of studied patients was 60.02 ± 23.1 years. 

Based on the results, 51 (63.7%),46 (57.5%),35 (43.8%),49 (61.3%),48 (60%), 56 (70%), 48 (60%), 35 (43.8%), 57 (71.3%), 60 (75%), and 35 (43.8 %) were sensitive to amikacin, cefepime, ciprofloxacin, gentamicin, levofloxacin, piperacillin, piperacillin/tazobactam, ceftazidime, meropenem, imipenem, and tobramycin respectively. Thus, the prevalence of multidrug-resistant (MDR) phenotype and non-MDR isolates was 44 (55%), and 36 (45%) respectively. 

### Biofilm production assay

The results of the biofilm formation test showed that all strains of *Pseudomonas aeruginosa* studied had the ability to form biofilm.

### Tracking of algD, pslD, pelF genes

We found that 71(88.8%),61 (76.3%), and 77 (96.3%) carried *algD*, *pelF*, and *pslD* genes, respectively. Among 80 *Pseudomonas aeruginosa* 68.7%(n=55) of isolates carried all three *"algD+, pslD+, pelF+*" genes, while 2.5%(n=2) isolates had no "*algD-, pslD−, pelF*−" genes. 

### The relationship between MDR and the presence of genes

The chi-square test showed a significant relationship between the presence of *pslD, pelF*, and *algD* genes and MDR (P=0.001) (Table 2). So, most isolates carrying *pslD, pelF*, and *algD* genes were MDR.

### The relationship between different variables and the existence of the studied genes

Based on the results of the ANOVA test (p ˃0.05), this study concluded that there is no significant correlation between the age of patients and the presence of the *algD, pelF, and pslD* genes. There was no significant correlation between the gender of patients and the presence of the *algD, pelF, and pslD *genes (p ˃0.05) in the chi-square test. Therefore, the chi-square test did not show a significant relationship between the presence of the *algD*, *pelF*, and *pslD* genes with the sample type (p ˃0.05) (Table 3).

In order to investigate the effect of age, gender and sampling location on the presence of each gene, a multiple logistic regression model was used. The results of this modeling for all three genes are shown in the table below. The results obtained from logistic regression modeling showed that the three variables of age, gender and sampling location did not affect the presence or absence of each gene (Table 4).

**Fig. 1:**
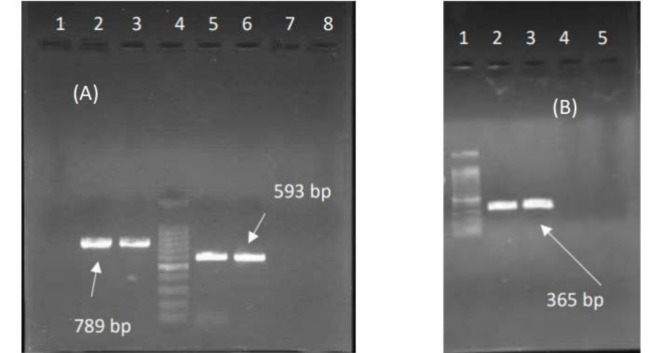
Electrophoresis of the PCR product of the studied genes. (A): PCR product of* algD*, *pelF*; Lane 1: Control negative for *pelF*, Lane 2: Clinical isolate for *pelF*, Lane 3: Control Positive for *pelF*, Lane 4: ladder 100 bp, Lane 5: Positive Control for *algD,* Lane 6: Clinical isolate for *algD*, Lane 7: Control negative for *algD.* (B): PCR product of* pslD*; Lane 1: ladder 100 bp, Lane 2: Positive Control, Lane 3,4: clinical isolates, Lane 5: Control negative.

**Table 2:** The relationship between MDR and the presence of genes

Genes	MDR N (%)	Non-MDR N (%)	P -value
*algD*	44(62.0)	27(38)	0.001
*pelF*	42(68.9)	19(31.1)	0.001
*pslD*	44(57.1)	33(42.9)	0.001

**Table 3:** The relationship between variables and *alg D, psl D, pel F* genes

Variables		*algD*			*pelF*			*pslD*		
		Mean	SD	P -value	Mean	SD	P -value	Mean	SD	P -value
Age		60.21	22.618	0.841	62.18	24.018	0.252	60.16	23.465	0.799
		**N (%)**		**P -value**	**N (%)**		**P -value**	**N (%)**		**P -value**
Sex	**Male**	40(56.3)		0.726	35(57.4)		0.999	43(55.8)		0.258
	**Female**	31(43.7)			26(42.6)			34(44.2)		
		**N (%)**		**P -value**	**N (%)**		**P -value**	**N (%)**		**P -value**
Sample type	**Urine**	31(43.7)		0.691	29(47.5)		0.206	36(46.8)		0.268
	**Wound**	12(16.9)			9(14.8)			12(15.6)		
	**Sputum**	19(26.8)			17(27.9)			19(24.7)		
	**Secretions**	4(5.6)			2(3.3)			4(5.2)		
	**Blood**	5(7.0)			4(6.6)			6(7.8)		

**Table 4:** The effect of age, gender, and sample type variables on the presence of genes

P-value	Variables	genes
0.755	**Age**	***algD***
0.507	**Sex**	
0.992	**Sample type**	
0.078	**Age**	***pelF***
0.591	**Sex**	
0.612	**Sample type**	
0.471	**Age**	***pslD***
0.999	**Sex**	
0.993	**Sample type**	

## Discussion

In the present study, investigating antibiotic resistance and sensitivity of clinical isolates of *Pseudomonas aeruginosa* showed that the highest sensitivity was related to imipenem and meropenem, and the highest resistance was related to ceftriaxone and cefotaxime. Additionally, 55% of the isolates were MDR, which poses a significant challenge in the treatment of infections caused by this bacterium. Khan et al. (2020) reported a 50% rate of multidrug resistance in *Pseudomonas aeruginosa* isolates, which is in agreement with our findings. The study also reported a high level of sensitivity to imipenem and meropenem (18). In 2020, Sebola et al. conducted a study that revealed that *Pseudomonas aeruginosa* isolates exhibited the highest sensitivity to imipenem and meropenem and the highest resistance to ceftriaxone and cefotaxime (19). On the other hand, a study conducted in Iran showed that 93% of *Pseudomonas aeruginosa* isolates had MDR (20), which is not consistent with our study. On the other hand, in another study in Iran, the rate of MDR was reported as 40% (21). By comparing the results of our study with other research, the difference in antibiotic sensitivity and resistance of clinical isolates is probably in terms of the difference in the pattern of antibiotic use and different treatment protocols in different geographical areas at different times.

One of the challenges of bacterial therapy is antibiotic resistance to conventional antibiotics, which is linked to biofilm formation (22). *Pseudomonas aeruginosa* biofilm matrix is made up of *algD, pelF, *and* pslD* genes (23). In our investigation, we looked at the frequency of these genes and how they correlated with the frequency of MDR isolates. In Rajabi et al.'s study, the frequency of *algD*, *pelF*, and *pslD* genes in* Pseudomonas aeruginosa* *isolated* from Sanandaj 2020-2021, west of Iran, was reported at 78.6, 70.5, and 36.6%, respectively (24). The frequency of *algD*, and *pelF* is consistent with our results. In Banar et al. study, the frequencies of these *pelF, algD,* and *pslD* genes in *Pseudomonas aeruginosa* isolated from burn wounds 2013-2014 were 93%, 100%, and 54.6%, respectively (17). In the study of Ghadaksaz et al., which was conducted to investigate virulence factors related to biofilm among *Pseudomonas aeruginosa* isolates, the frequency of *algD* gene was reported as 87.5% (25), which is in agreement with our results.

According to the results of the Colvin et al. investigation, Pel polysaccharide and its linked gene (*pelF*) play an important role in biofilm formation and antibiotic resistance in *Pseudomonas aeruginosa* (26). The current work looked at the link between the *algD, pelF, and pslD* genes and MDR in *Pseudomonas aeruginosa*. The results revealed that the presence of the *algD* gene is substantially associated with the formation of MDR, indicating that isolates lacking this gene were not MDR. However, 62% of strains with the *algD* gene were MDR (P < 0.05). Thus, the *pelF* gene was reported in 68.9% of MDR isolates and similar results were reported for the *pslD* gene. These results indicated the key role of these genes in the development of MDR and can be used as biological markers to identify resistant isolates. The significant relationship between the presence of these genes and MDR can be in terms of their role in biofilm formation, which is known to be one of the main factors in increasing antibiotic resistance in *Pseudomonas aeruginosa*.

The limitations of the present study included the limited time and the focus of the research on a specific geographical area. In this research, by identifying the pattern of antibiotic resistance and the results of gene analysis, it is possible to infection control centers of hospitals in the field of antibiotic prescription.

## Conclusion

The findings of this study, by examining the *algD, pelF*, and *pslD* genes, highlight the need for new therapeutic techniques for *Pseudomonas aeruginosa* infections. In addition, further research is needed to study the precise processes of the influence of these genes on antibiotic resistance.

## Notes on Contributors

M.A. conducted the literature search, selected the studies, and prepared the manuscript. M.P. conducted the literature search and edited the manuscript. A.T. prepared the manuscript and extracted data. M.A.A. wrote and edited the manuscript. F.Z. edited the manuscript and extracted data. S.E. was responsible for study selection and data extraction. M.H. evaluated data and conceptualized the manuscript. All authors read the article in full and approved it.

## Data Availability

There is no additional data separate from available in cited references.
